# Weft, Warp, and Weave: The Intricate Tapestry of Calcium Channels Regulating T Lymphocyte Function

**DOI:** 10.3389/fimmu.2013.00164

**Published:** 2013-06-24

**Authors:** Kyla D. Omilusik, Lilian L. Nohara, Shawna Stanwood, Wilfred A. Jefferies

**Affiliations:** ^1^Michael Smith Laboratories, University of British Columbia, Vancouver, BC, Canada; ^2^Centre for Blood Research, University of British Columbia, Vancouver, BC, Canada; ^3^The Brain Research Centre, University of British Columbia, Vancouver, BC, Canada; ^4^Department of Microbiology and Immunology, University of British Columbia, Vancouver, BC, Canada; ^5^Department of Medical Genetics, University of British Columbia, Vancouver, BC, Canada; ^6^Department of Zoology, University of British Columbia, Vancouver, BC, Canada

**Keywords:** calcium, T cell, calcium channels, L-type calcium channels, T cell signaling

## Abstract

Calcium (Ca^2+^) is a universal second messenger important for T lymphocyte homeostasis, activation, proliferation, differentiation, and apoptosis. The events surrounding Ca^2+^ mobilization in lymphocytes are tightly regulated and involve the coordination of diverse ion channels, membrane receptors, and signaling molecules. A mechanism termed store-operated Ca^2+^ entry (SOCE), causes depletion of endoplasmic reticulum (ER) Ca^2+^ stores following T cell receptor (TCR) engagement and triggers a sustained influx of extracellular Ca^2+^ through Ca^2+^ release-activated Ca^2+^ (CRAC) channels in the plasma membrane. The ER Ca^2+^ sensing molecule, stromal interaction molecule 1 (STIM1), and a pore-forming plasma membrane protein, ORAI1, have been identified as important mediators of SOCE. Here, we review the role of several additional families of Ca^2+^ channels expressed on the plasma membrane of T cells that likely contribute to Ca^2+^ influx following TCR engagement, particularly highlighting an important role for voltage-dependent Ca^2+^ channels (Ca_V_) in T lymphocyte biology.

In the body’s steady-state, a pool of T lymphocytes that express a diverse T cell receptor (TCR) repertoire is maintained in the periphery. In the event of an infection, T lymphocytes, through their TCR, recognize the infectious antigen and are activated and subsequently induced to proliferate and differentiate into effector cells capable of clearing the pathogen. Key components of the signaling events mediating T lymphocyte development, differentiation, homeostasis, effector function, and cell death are the universal second messenger calcium (Ca^2+^) and the Ca^2+^ channels that regulate the intracellular Ca^2+^ levels (Smith-Garvin et al., 2009).

The activation of a T cell occurs when its TCR recognizes cognate antigen presented on major histocompatibility complex (MHC) by an antigen processing cell. In primary immune responses, this is the function of dendritic cell (DC). DCs take up soluble and particulate antigen as well as cellular debris by phagocytosis, endocytosis, or macropinocytosis and degrade them in endolysosomal compartments where liberated foreign antigens, usually peptides, are subsequently loaded onto MHC-I or MHC-II molecules that cycle to the plasma membrane. Here, the MHC/foreign antigen complex is recognized by a cognate TCR expressed on a specific T lymphocyte (Vyas et al., 2008). A series of signaling events ensue following ligation of the TCR. Ca^2+^ is critical to the TCR signaling processes. TCR engagement triggers an increase in intracellular Ca^2+^ levels resulting from the activation of phospholipase Cγ1 (PLCγ1) and the associated hydrolysis of phosphatidylinositol-3,4-bisphosphate (PIP_2_) into inositol-1,4,5-trisphosphate (IP_3_) and diacylglycerol (DAG). IP_3_ binds to IP_3_ receptors (IP_3_R) in the endoplasmic reticulum (ER) causing release of ER Ca^2+^ stores into the cytoplasm. During the event of store-operated Ca^2+^ entry (SOCE), depletion of ER Ca^2+^ stores triggers a sustained influx of extracellular Ca^2+^ through Ca^2+^ release-activated Ca^2+^ (CRAC) channels in the plasma membrane (Hogan et al., 2010).

The sustained increase in intracellular Ca^2+^ results in the activation of signaling molecules and transcription factors that induce expression of genes required for T cell activation, proliferation, differentiation, and effector function. In T cells, Ca^2+^ can activate a variety of targets including the serine/threonine phosphatase calcineurin and its transcription factor target nuclear factor of activated T cells (NFAT), Ca^2+^-calmodulin-dependent kinase (CaMK) and its target cyclic AMP-responsive element-binding protein (CREB), myocyte enhancer factor 2 (MEF2) targeted by both calcineurin and CaMK, and nuclear factor kappa B (NFκB) (Oh-Hora, 2009). The best studied downstream effect of Ca^2+^ is the calcineurin-NFAT pathway. Increased Ca^2+^ levels promote the binding of Ca^2+^ to calmodulin inducing a conformational change that allows calmodulin to bind and activate calcineurin. Calcineurin dephosphorylates serines in the amino-terminus of NFAT exposing a nuclear localization signal. This results in the transport of NFAT into the nucleus, where NFAT can interact with other transcription factors, integrating signaling pathways, and inducing gene expression patterns dependent on the context of the TCR signaling (Hogan et al., 2003; Macian, 2005; Smith-Garvin et al., 2009). Ca^2+^ has also been proposed to regulate the Ras/mitogen-activated protein kinase (MAPK) pathway in T cells. RasGRP that activates Ras not only has a DAG binding domain but also has a pair of EF-hand motifs that can directly bind Ca^2+^ (Cullen and Lockyer, 2002). Through this interaction, activation and membrane localization of Ras guanyl nucleotide-releasing protein (RasGRP) is influenced. Upon weak TCR stimulation, RasGRP localizes to the Golgi membrane whereas strong TCR signaling results in recruitment to the plasma membrane. The site of activation may play a role in what extracellular-signal-regulated kinase (ERK) can target downstream thereby contributing to differential signaling dependent on the stimulus (Teixeiro and Daniels, 2010).

There are several families of channels expressed on the plasma membrane of T lymphocytes (Kotturi et al., 2006) that may play important roles in Ca^2+^ entry (Figure 1). Recently, through genome wide high-throughput RNA interference screens and analysis of patients with severe combined immunodeficiency (SCID), a pore-forming plasma membrane protein, ORAI1 (Feske et al., 2006; Vig et al., 2006; Zhang et al., 2006), and an ER Ca^2+^ sensing molecule, stromal interaction molecule 1 (STIM1) (Liou et al., 2005; Roos et al., 2005), have been identified as the classically defined CRAC channel. Transient receptor potential (TRP) channels have also been the focus of much attention and have been reported to be activated by store depletion in T cells. IP_3_ receptors (IP_3_R), similar to the ER-associated Ca^2+^ channels, have been shown to be expressed at the plasma membrane of T cells. In addition, T cell expressed adenosine triphosphate (ATP) responsive purinergic P2 (P2X) receptors and glutamate mediated *N*-methyl-d-aspartate (NMDA) activated receptors have shown significant Ca^2+^ permeability. Finally, voltage-dependent Ca^2+^ channels (Ca_V_), the focus of this review, have been identified to play a crucial function in T cells (Omilusik et al., 2011).

**Figure 1:**
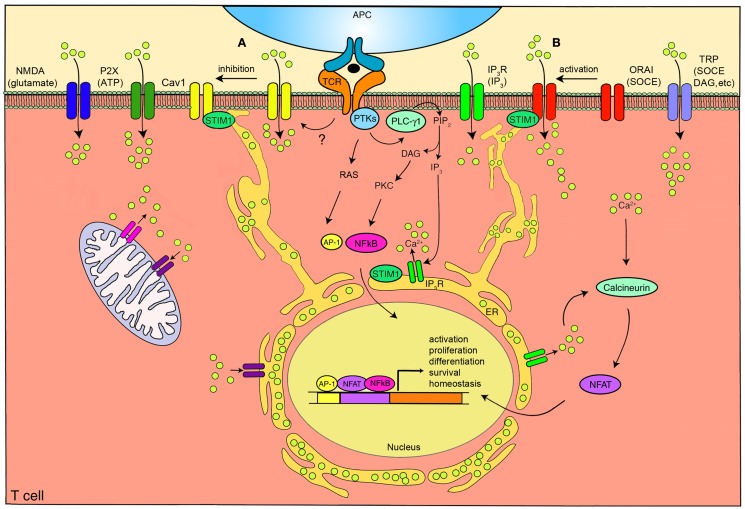
**The calcium channels in T cells**. T cell receptor (TCR) engagement by a peptide-MHC on an antigen presenting cell (APC), induces protein tyrosine kinases (PTKs) to activate phospholipase C-γ1 (PLC-γ1), which cleaves phosphatidylinositol 4,5-bisphosphate (PIP_2_) from plasma membrane phospholipids to generate diacylglycerol (DAG) and inositol-1,4,5-trisphosphate (IP_3_). Elevated levels of IP_3_ in the cytosol leads to the release of Ca^2+^ from IP_3_R Ca^2+^ channels located in the endoplasmic reticulum (ER). Ca^2+^ depletion from the ER induces Ca^2+^ influx from the extracellular space through the plasma membrane channel, ORAI1. Several auxiliary channels also operate during TCR-mediated Ca^2+^ signaling. These include plasma membrane IP_3_R activated by the ligand IP_3_, transient receptor potential (TRP) channels that can be operated by DAG and store-operated Ca^2+^ entry (SOCE), adenosine triphosphate (ATP) responsive purinergic P2 (P2X) receptors, glutamate mediated *N*-methyl-D-aspartate activated (NMDA) channels, and voltage-dependent Ca^2+^ channels (Ca_V_1) that may be regulated through TCR signaling events. The mitochondria also control cytoplasmic Ca^2+^ levels. Increase in intracellular Ca^2+^ results in activation of calmodulin-calcineurin pathway that induces nuclear factor of activated T cells (NFAT) nuclear translocation and transcription of target genes to direct T cell homeostasis, activation, proliferation, differentiation, and survival. Within this complex network of Ca^2+^ signaling, a model of the reciprocal regulation of Ca_V_1 and ORAI1 in T cells has been proposed. **(A)** Low-level TCR signaling through interactions with self-antigens (i.e., self-peptides/self-MHC molecules) may result in Ca_V_1 (particularly Ca_V_1.4) activation and Ca^2+^ influx from outside the cell. This allows for filling of intracellular stores and initiation of a signaling cascade to activate a pro-survival program within the naïve T cell. Stromal interaction molecule 1 (STIM1) is not activated in this scenario and, consequently, ORAI1 remains closed. **(B)** Strong TCR signaling through engagement by a foreign peptide-MHC induces the downstream signaling events that result in ER Ca^2+^ store depletion and STIM1 accumulation in puncta in regions of the ER near the plasma membrane allowing interactions with Ca^2+^ channels. ORAI1 enhances STIM1 recruitment to the vicinity of Ca_V_1 channels. Here, STIM1 can activate ORAI1 while inhibiting Ca_V_1. PKC, protein kinase C. AP-1, activating protein-1. NFκB, nuclear factor kappa B. Yellow circles, Ca^2+^.

## ORAI and STIM

The discovery of the pore-forming plasma membrane proteins, ORAI1 and homologs ORAI2 and ORAI3, and the ER Ca^2+^ sensors, STIM1 and STIM2, has led to the development of a well-established paradigm of their coordinated action (Hogan et al., 2010; Feske et al., 2012; Srikanth and Gwack, 2012). TCR engagement triggers the generation of IP_3_ and the subsequent activation of IP_3_Rs that mediate the release of Ca^2+^ from the ER. The ER transmembrane protein, STIM1, can sense the depletion of Ca^2+^ stores. STIM1 exists as a monomer when Ca^2+^ is present, and its conformation is stabilized through an interaction between its luminal EF-hand domain and sterile α-motif (SAM). When ER Ca^2+^ stores are depleted, the EF-SAM domain interaction in STIM1 becomes unstable resulting in the oligomerization of STIM1 molecules (Park et al., 2009; Stathopulos et al., 2009). STIM1 oligomers accumulate in puncta in regions of ER 10–25 nm beneath the plasma membrane (Liou et al., 2005, 2007; Wu et al., 2006a). Here, ORAI1 at the plasma membrane can interact with STIM1 (Luik et al., 2006; Xu et al., 2006). ORAI1 has been suggested to exist as a dimer in the plasma membrane and upon STIM1 interaction forms tetramers that can function to import Ca^2+^ (Penna et al., 2008).

Analyses of ORAI1 and STIM1 deficiency in human patients, that initially led to the identification of ORAI (Feske et al., 2006), as well as in mouse models, have validated their physiological role in T cell activation. In humans, loss of functional ORAI1 or STIM1 results in SCID (Partiseti et al., 1994; Le Deist et al., 1995; Feske et al., 2001, 2005, 2006; Picard et al., 2009). While lymphocyte numbers are normal in these patients, impaired SOCE leaves T cells with diminished ability to proliferate and produce cytokines upon activation. Analogous phenotypes are observed in animal models. In *ORAI1*^−*/*−^ and *STIM1*^−*/*−^ mice, thymic development of conventional TCRαβ T cells appears normal. However, impaired selection of agonist-selected T cells, T regulatory cells (T_reg_), invariant natural killer T cells and TCRαβ^+^ CD8αα^+^ intestinal intraepithelial lymphocytes, owing to a defect in IL-2 or IL-15 signaling has been noted in STIM1- and STIM2-deficient mice (Oh-Hora et al., 2013). ORAI1-deficiency causes a moderate reduction in SOCE and subsequent cytokine production in T cells (Gwack et al., 2008; Vig et al., 2008). STIM1-deficient T cells have no CRAC channel function or SOCE, no subsequent activation of NFAT transcription factor and, as a result, have impaired cytokine secretion (Oh-Hora et al., 2008). This impacts T cell responses and, consequently, confers protection from experimental autoimmune encephalomyelitis (EAE) due to poor generation of Th_1_/Th_17_ responses (Schuhmann et al., 2010).

Interestingly, STIM-deficiency is also associated with lymphoproliferative and autoimmune diseases. In SCID patients, this is seen as lymphadenopathy (enlarged lymph nodes) and hepatosplenomegaly (enlarged liver and spleen) as well as autoimmune hemolytic anemia and thrombocytopenia resulting from immune responses directed against the red blood cells and platelets, respectively (Picard et al., 2009). It has been suggested that this autoimmunity observed in STIM1-deficient patients is a consequence of the reduced T_reg_ cell numbers found in the periphery (Feske, 2009; Picard et al., 2009). Similarly, mice lacking both STIM1 and STIM2 experienced autoimmune and lympho-myeloproliferative syndromes again due to a severe reduction in T_reg_ number in the thymus and secondary lymphoid organs and impaired T_reg_ suppressive function (Oh-Hora et al., 2008). This T_reg_ deficiency is presumably a result of poor Ca^2+^/NFAT-dependent induction of Foxp3 expression (Wu et al., 2006b; Oh-Hora et al., 2008; Tone et al., 2008). Together, these studies highlight the importance of ORAI1/STIM1 in T cell activation and immune tolerance.

T cells also express family members ORAI2 and ORAI3 that exhibit similar structure to ORAI1. ORAI2 and ORAI3 form Ca^2+^-permeable ion pores; however, these channels differ in their pharmacology, ion selectivity, activation kinetics, and inactivation properties in comparison to ORAI1 (Lis et al., 2007). Overexpression of ORAI2 or ORAI3 with STIM1 can result in Ca^2+^ currents similar but not identical to the CRAC current (DeHaven et al., 2007; Lis et al., 2007). However, ORAI2’s contribution to Ca^2+^ signaling in differentiated T cells is questionable as overexpression of ORAI2 in ORAI1^−*/*−^ T cells does not restore SOCE (Gwack et al., 2008). ORAI2 expression is high in naïve T cells and is down regulated upon activation; therefore, ORAI2 may have a major role in development or peripheral homeostasis (Gwack et al., 2008; Vig et al., 2008). ORAI3 has been shown to form pentamers with ORAI1 to make up the arachidonate-regulated Ca^2+^-selective (ARC) channels (Mignen et al., 2009). These channels are activated by arachidonic acid rather than store-depletion and require plasma membrane localized STIM1 for their regulation (Mignen et al., 2007). Their role in T cells has yet to be determined.

STIM2 with 42% sequence similarity to STIM1 is also found in T cells. While STIM1 has relatively high and constant expression and can be found to some extent in the plasma membrane as well as the ER, STIM2 is expressed at low levels in naïve T cells but is upregulated upon TCR activation and is exclusively localized to the ER (Williams et al., 2001; Soboloff et al., 2006). Like STIM1, STIM2 functions as an ER Ca^2+^ sensor and is able to mediate SOCE in lymphocytes. Nevertheless, STIM2 does not seem to serve a redundant purpose as its overexpression only partially rescues Ca^2+^ influx deficiency in STIM1^−*/*−^ T cells (Brandman et al., 2007; Oh-Hora et al., 2008). Upon Ca^2+^ store depletion, STIM2 also oligomerizes and localizes to puncta at ER-plasma membrane contacts; however, STIM2 detects smaller decreases in ER Ca^2+^ concentration and forms multimers with slower kinetics than STIM1 with some STIM2 already activated in resting cells with replete Ca^2+^ stores (Soboloff et al., 2006; Brandman et al., 2007). This fits with the established role for STIM2 in regulating basal Ca^2+^ influx and stabilizing cytosolic and ER Ca^2+^ levels in resting cells (Brandman et al., 2007). It also explains the fact that STIM2-deficiency has minimal effect on the initial Ca^2+^ entry but impairs the ability of T cells to maintain nuclear translocation of NFAT and cytokine production (Oh-Hora et al., 2008). Where STIM1 readily senses ER Ca^2+^ store depletion and can initiate SOCE, STIM2 remains active in higher Ca^2+^ levels when stores are refilling and can sustain the response (Oh-Hora, 2009).

Although the details of the ORAI-STIM pathway have been the subject of a large amount of recent work, this scheme does not account for the involvement of other currents mediated by additional plasma membrane Ca^2+^ channels that have been shown to be expressed and function in T cells (Kotturi et al., 2003; Kotturi and Jefferies, 2005; Omilusik et al., 2011), nor does it allow for differential patterns in Ca^2+^ response between T cell subsets (Fanger et al., 2000; Weber et al., 2008; Robert et al., 2011). Immunologists are only beginning to acknowledge, accept, and integrate these channels into the pantheon of functions mediated by T cells. Therefore, incorporating multiple Ca^2+^ channels into a comprehensive model is essential for the complete understanding of Ca^2+^ signaling in T cells.

## Important Additional Ca^2+^ Channels in T Lymphocytes

### IP_3_ receptors

The IP_3_Rs, similar to those found in the ER, have been suggested to function as Ca^2+^ channels at the plasma membrane (Khan et al., 1992; Kotturi et al., 2006). IP_3_ dissipates rapidly after TCR engagement; therefore, IP_3_ induced activation of plasma membrane receptors would only contribute to short-term Ca^2+^ signaling (Kotturi et al., 2006). Alternatively, it was suggested that IP_3_Rs in the ER, known to bind IP_3_ to deplete ER Ca^2+^ stores, change conformation upon ER store depletion, and signal to surface IP_3_Rs to open (Berridge, 1993). IP_3_Rs have been identified on the cell surface of cultured T cells (Khan et al., 1992; Tanimura et al., 2000). However, IP_3_-induced Ca^2+^ currents across the plasma membrane could not be detected (Zweifach and Lewis, 1993). As an alternate function based on the numerous protein binding sites present in the modulatory domain of the channel, IP_3_Rs have been proposed to operate at the plasma membrane as scaffolds (Patterson et al., 2004). Further work is required to clearly fit the IP_3_R into the Ca^2+^ signaling network in T cells.

### Transient receptor potential channels

The first TRP family member was discovered in *Drosophila* and was found to have a role in visual transduction (Montell and Rubin, 1989). Subsequently, 28 mammalian TRP channel proteins have been identified. These are grouped into six subfamilies based on amino acid sequence similarities: the classical TRPs (TRPCs) that are most similar to *Drosophila* TRP; the vanilloid receptor TRPs (TRPVs); the melastatin TRPs (TRPMs); the mucolipins (TRPMLs); the polycystins (TRPPs); and ankyrin transmembrane protein 1 (TRPA1) (Clapham et al., 2003; Montell and Rubin, 1989). The six transmembrane domain TRP channels form pores that are permeable to cations including Ca^2+^ (Owsianik et al., 2006). Various TRP channel family members have been shown to be expressed in cultured or primary T cells (Schwarz et al., 2007; Oh-Hora, 2009; Wenning et al., 2011).

Before the discovery of ORAI1 and STIM1, TRP channels were investigated as candidates for the CRAC channel. The TRPV6 channel is highly permeable to Ca^2+^ and has been shown to be activated by store-depletion (Cui et al., 2002). In addition, when a dominant-negative pore-region mutant of TRPV6 was expressed in Jurkat T cells, the CRAC current was diminished (Cui et al., 2002). However, in subsequent studies, the CRAC channel inhibitor, BTP2, had no effect on TRPV6 channel activity (Zitt et al., 2004; He et al., 2005; Schwarz et al., 2006) and the role of TRPV6 as a CRAC channel could not be confirmed (Voets et al., 2001; Bodding et al., 2002). TRPC3 channels were also under consideration as CRAC channels following the discovery that Jurkat T cell lines with mutated TRPC3 channels had reduced Ca^2+^ influx following TCR stimulation. This impairment could be overcome by overexpression of a wild-type TRPC3 (Fanger et al., 1995; Philipp et al., 2003). Furthermore, siRNA knockdown of TRPC3 expression in human T cells resulted in reduced proliferation following TCR stimulation (Wenning et al., 2011). However, while TRPC3 has been shown to be activated in response to store-depletion (Vazquez et al., 2001), the major stimulus gating TRPC3 seems to be DAG (Hofmann et al., 1999).

Although not store-operated, the TRPM2 channel in T cells has also been examined. TRMP2 is a non-selective Ca^2+^ channel that is activated by the intracellular secondary messengers ADP-ribose (ADPR), nicotinamide adenine dinucleotide (NAD^+^), hydrogen peroxide (H_2_O_2_), and cyclic ADPR (Perraud et al., 2001; Hara et al., 2002; Massullo et al., 2006). It has been proposed that activation of T cells can increase endogenous ADPR levels in T cells which results in Ca^2+^ entry through TRPM2 and subsequent induction of cell death demonstrating that TRPM2 can contribute to some degree to Ca^2+^ signaling in T cells (Gasser et al., 2006). Recently, the TRPM2 channels have been implicated in T cell effector function. CD4^+^ T cells from TRPM2-deficient mice were shown to have reduced ability to proliferate and secrete cytokines following TCR activation. Furthermore, TRPM2-deficient mice had decreased inflammation and demyelinating spinal cord lesions in an EAE model (Melzer et al., 2012). Although important to T cell function, the current role of TRP receptors in Ca^2+^ signaling is still under investigation.

### ATP-responsive purinergic P2 receptors (P2X)

The P2X receptors are ATP-gated ion channels that permit the influx of extracellular cations including Ca^2+^ ions (reviewed in Junger, 2011). Four family members in particular, P2X1, P2X2, P2X4, and P2X7, have been associated with T cells and may serve to amplify the TCR signal to ensure antigen recognition and T cell activation through an autocrine feedback mechanism (Bours et al., 2006; Yip et al., 2009; Woehrle et al., 2010; Junger, 2011). Upon TCR engagement, ATP is released through Pannexin 1 hemichannels that localize to the immunological synapse where they release ATP that acts on the P2X channels to promote Ca^2+^ influx and enhance signaling (Filippini et al., 1990; Schenk et al., 2008; Yip et al., 2009). In particular, P2X1, 4, and 7 have been shown to contribute to the increase in intracellular Ca^2+^, NFAT activation, proliferation, and IL-2 production in murine and human T cells following stimulation (Baricordi et al., 1996; Schenk et al., 2008; Yip et al., 2009; Woehrle et al., 2010). Initial analysis of P2X7 receptor-deficient mice revealed no major defects in T cell development (Solle et al., 2001). However, additional studies did identify a deficiency in T_reg_ stability and function as well as Th_17_ differentiation (Schenk et al., 2011). Also, T cells from C57Bl/6 mice with a natural mutation in the P2X7 gene that reduces ATP sensitivity have been shown to produce reduced amounts of IL-2 following stimulation compared to Balb/c mice with a fully functional receptor further delineating a role for P2X receptors in T cell function (Adriouch et al., 2002; Yip et al., 2009). Likewise, in two models of T cell-dependent inflammation, treatment with a P2XR antagonist impeded the development of colitogenic T cells in inflammatory bowel disease and induced unresponsiveness in anti-islet TCR transgenic T cells in diabetes (Schenk et al., 2008). Therefore, it is clear that P2X channels are playing an important role in T cell Ca^2+^ signaling; however, the specific mechanistic details of how they fit into shaping the T cell Ca^2+^ environment need further exploration.

### *N*-methyl-d-aspartate activated receptors

The NMDA receptors are a class of ligand-gated glutamate ionotropic receptors found in the central nervous system that play a crucial role in neuronal function. These receptors are heterotetramers composed of two subunits, NR1 and NR2, that form an ion channel which is highly permeable to K^+^, Na^+^, and Ca^2+^ (Boldyrev et al., 2012). Ca^2+^ entry through the receptors into the cell occurs when the NMDA receptors are activated by binding to their ligands, glutamate and glycine. In neurons, this allows for long-lasting memory formation (Boldyrev et al., 2012). Interestingly, NMDA receptors have been shown to be expressed on rodent and human T cells and contribute to the increase in intracellular Ca^2+^ level following T cell activation (Lombardi et al., 2001; Boldyrev et al., 2004; Miglio et al., 2005, 2007; Mashkina et al., 2007, 2010). Zainullina et al. (2011) further demonstrated that activation of T cells with thapsigargin, an inhibitor of a Ca^2+^-ATPase of the ER that induces Ca^2+^ store depletion and activation of plasma membrane Ca^2+^ channels, in the presence of an NMDA receptor antagonist did not affect the movement of Ca^2+^ from intracellular stores. However, it reduced the influx of Ca^2+^ from the extracellular space suggesting that NMDA receptors participate in SOCE, at least to some degree. In this scenario, the NMDA receptors may be mainly contributing to Ras/Rac-dependent signaling in T cells following TCR engagement (Zainullina et al., 2011). Analogous to neuronal synapses, a recent study of thymocytes showed that upon TCR stimulation, NMDA receptors localize to the immunological synapse (Affaticati et al., 2011). Here, DCs rapidly release glutamate that activates the NMDA receptors on the T cells contributing to the increase in intracellular Ca^2+^ concentration. It is suggested that glutamate signaling through these receptors may participate in negative selection in the thymus by inducing apoptosis in thymocytes while it may influence proliferation in peripheral T cells (Affaticati et al., 2011). Further studies are required to determine the role glutamate plays in shaping the Ca^2+^ signal in T cells.

### Voltage-dependent Ca^2+^ channels

Ca_V_ channels function typically in excitable cells such as nerve, muscle, and endocrine cells where they open in response to membrane depolarization to allow Ca^2+^ entry (Buraei and Yang, 2010). The Ca_V_ channels were initially classified based on the voltage required for activation into the subgroups high-voltage activated (HVA) and low-voltage activated (LVA) channels. Further analysis of the Ca_V_ channels allowed for additional classification of the channels into groups with distinct biophysical and pharmacological properties: T (tiny/transient)-, N (neuronal)-, P/Q (Purkinje)-, R (toxin-resistant)-, L (long-lasting)-type channels (Lacinova, 2005; Buraei and Yang, 2010).

The Ca_V_ channels are heteromultimeric protein complexes composed of five subunits: α_1_, α_2_, β, δ, and γ. The α_2_ and δ subunits are linked together through disulfide bonds to form a single unit referred to as α_2_δ. The α_1_ subunit of the channel is the pore-forming component responsible for the channel’s unique properties while the α_2_δ, β, and γ subunits regulate the structure and activity of α_1_ (Buraei and Yang, 2010). The α_1_ subunit consists of four homologous repeated motifs (I–IV) each composed of six transmembrane segments (S1–S6) with a re-entrant pore-forming loop (P-loop) between S5 and S6. The P-loop contains four highly conserved negatively charged amino acids responsible for selecting and conducting Ca^2+^ while the S6 segments form the inner pore (Buraei and Yang, 2010). The S4 segments are positively charged and constitute the voltage sensor. The pore opens and closes through voltage-mediated movement of this sensor (Lacinova, 2005).

Ten mammalian α_1_ subunits are divided into three subfamilies based on similarities in amino acid sequence. The Ca_V_1 family contains L-type channels; the Ca_V_2 family consists of N-, P/Q-, and R-type channels; and the Ca_V_3 family are T-type channels (Buraei and Yang, 2010). Initially, “voltage-operable” current seemingly activated by TCR engagement or store depletion with electrophysiological properties different than the CRAC current in the plasma membrane of Jurkat T cells was identified (Densmore et al., 1992, 1996). Subsequently, numerous pharmacological and genetic studies have demonstrated the existence of Ca_V_1 or L-type channels in T cells (Table 1). The Ca_V_1 channels exist as four isoforms: Ca_V_1.1, Ca_V_1.2, Ca_V_1.3, and Ca_V_1.4. In excitable cells, L-type Ca^2+^ channels require high-voltage activation and have slow current decay kinetics. They have a unique sensitivity to 1,4-dihydropyridines (DHPs), a wide drug class that can either activate (for example: Bay K 8644) or inhibit (for example: nifedipine) the activity of the channel (Lacinova, 2005).

**Table 1:** **Ca_V_1 Ca^2^^+^ channel expression in T cells**.

Subtype	Distribution	Analysis	Reference
Ca_V_1.1	Mouse CTLs	Protein	Matza et al. (2009)
	Mouse effector CD8^+^ T cells	mRNA (PCR); protein	Jha et al. (2009)
	Mouse CD4^+^ T cells	mRNA (PCR); protein	Badou et al. (2006), Matza et al. (2008)
Ca_V_1.2	Human peripheral blood T cells; human Jurkat, MOLT-4, CEM T cell lines	mRNA (partial sequence); protein (truncated/full)	Stokes et al. (2004)
	Mouse CTLs	Protein	Matza et al. (2009)
	Mouse CD8^+^ T cells	mRNA (PCR)	Jha et al. (2009)
	Mouse CD4^+^ T cells	mRNA (PCR); protein	Badou et al. (2006), Matza et al. (2008)
	Mouse CD4^+^ Th2 cells	mRNA (sequence); protein	Cabral et al. (2010)
	Mouse BDC2.5 CD4^+^ T cells	mRNA (PCR)	Lee et al. (2008)
Ca_V_1.3	Human Jurkat T cell line	mRNA (partial sequence); protein (truncated)	Stokes et al. (2004)
		mRNA (PCR)	Colucci et al. (2009)
	Mouse CD8^+^ T cells	mRNA (PCR)	Jha et al. (2009)
	Mouse CD4^+^ Th2 cells	mRNA (sequence); protein	Cabral et al. (2010)
Ca_V_1.4	Human Jurkat T cell line; human spleen; human peripheral blood CD4^+^/CD8^+^ T cells	mRNA (sequence); protein	Kotturi et al. (2003), Kotturi and Jefferies (2005)
	Human spleen and thymus; rat spleen and thymus	mRNA (PCR); protein	McRory et al. (2004)
	Mouse T cells	mRNA (PCR); protein (truncated)	Omilusik et al. (2011)
	Mouse naïve CD8^+^ T cells	mRNA (PCR); protein	Jha et al. (2009)
	Mouse CD4^+^ T cells	mRNA (PCR)	Badou et al. (2006), Colucci et al. (2009)

Early studies suggesting that L-type Ca^2+^ channels contributed to T cell Ca^2+^ signaling relied on pharmaceutical analysis (Grafton and Thwaite, 2001; Kotturi et al., 2003; Gomes et al., 2004). These include *in vitro* experiments where the DHP antagonist nifedipine was shown to block proliferation of human T cells or peripheral blood mononuclear cells or impair increase in intracellular Ca^2+^following stimulation with mitogens (Birx et al., 1984; Gelfand et al., 1986; Dupuis et al., 1993). This effect of nifedipine seemed to be dose-dependent when T cells were stimulated in the presence of the immunosuppressive agent cyclosporine A (Marx et al., 1990; Padberg et al., 1990). In a resultant study performed by Kotturi et al. (2003), treatment of Jurkat T cells and human peripheral blood T cells with the DHP agonist Bay K 8644 was shown to increase intracellular Ca^2+^ levels and induce ERK 1/2 phosphorylation, while treatment with the DHP antagonist nifedipine blocked Ca^2+^ influx, ERK 1/2 phosphorylation, NFAT activation, IL-2 production, and T cell proliferation. At micromolar concentrations, DHPs can also affect the function of K^+^ channels and therefore conclusions drawn from these pharmaceutical studies (Grafton and Thwaite, 2001; Kotturi et al., 2003, 2006; Gomes et al., 2004) regarding contribution of Ca_V_1 to T cell function have been criticized (Wulff et al., 2003, 2004). However, inhibitory effects have been noted when DHP antagonists were used at concentrations well below those influencing K^+^ channels (Sadighi Akha et al., 1996; Kotturi et al., 2003) as well as with the more specific Ca_V_1 blocker, calciseptine, that also obstructs Ca^2+^ influx in T cells (de Weille et al., 1991; Matza and Flavell, 2009).

Subsequent genetic studies have confirmed the expression of L-type Ca^2+^ channels in T cells and have gone on to compare their structure to those found in excitable cells. Ca_V_1.4 was the first Ca_V_1 channel identified in T cells (Kotturi et al., 2003; Kotturi and Jefferies, 2005; Omilusik et al., 2011). The Ca_V_1.4 α_1_ subunit is encoded by the *CACNA1F* gene originally cloned from human retina (Fisher et al., 1997) where Ca_V_1.4 mediates Ca^2+^ entry into the photoreceptors promoting tonic neurotransmitter release (Strom et al., 1998). Kotturi et al. identified the Ca_V_1.4α_1_ subunit mRNA and protein in Jurkat T cells as well as in human peripheral blood T cells (Kotturi et al., 2003; Kotturi and Jefferies, 2005). This human lymphocyte form of Cav1.4 was shown to undergo alternative splicing, resulting in a protein smaller in size compared to a retinoblastoma version (Kotturi and Jefferies, 2005). Sequence analysis revealed that the Ca_V_1.4 expressed in human T cells exists as two novel splice variants (termed Ca_V_1.4a and Ca_V_1.4b) distinct from the retina (Kotturi and Jefferies, 2005). Ca_V_1.4a lacks exons 31, 32, 33, 34, and 37 resulting in deletions of transmembrane segments S3, S4, S5, and half of S6 in motif IV. As a result, the voltage sensor domain and part of the DHP binding site and EF-hand Ca^2+^ binding motif are deleted from the channel. While the removal of the voltage sensor may alter the voltage-gated activation of this channel, partial deletion of the DHP binding site may decrease the sensitivity of T cell-specific Ca_V_1.4 channels. This explained why large doses of DHP antagonists are required to completely block Ca^2+^ influx through Ca_V_ channels in T cells (Dupuis et al., 1993). Remarkably, the splice event caused a frameshift that changed the carboxy-terminus to a sequence that resembles (40% identity) the Ca_V_1.1 channel found in skeletal muscle (Kotturi and Jefferies, 2005). The second splice variant, Ca_V_1.4b, lacks exons 32 and 36 causing a deletion of the extracellular loop between S3 and S4 in motif IV. Ca_V_1.4b also has an early stop codon that prematurely truncates the channel. The voltage sensing motif is not spliced out; however, it has been proposed that removal of the extracellular loop may alter the voltage sensing function of this channel (Kotturi and Jefferies, 2005). Upon membrane depolarization, the S4 voltage sensor domain moves and this splicing event may leave the domain in a conformation that prevents S4 movement (Bezanilla, 2002; Jurkat-Rott and Lehmann-Horn, 2004). Since their discovery in T cells (Kotturi and Jefferies, 2005), alternative splice variants of other Ca_V_ channels have been found. Analogous structural changes have been subsequently noted for Ca_V_1.1 (Matza and Flavell, 2009) with one isoform similarly lacking the extracellular loop between S3 and S4 in motif IV that translated to shifted voltage sensitivity in muscle cells (Tuluc et al., 2009). These structural changes likely explain the insensitivity of T cell Ca_V_1 channels to be activated by cell depolarization and instead, gating in T cells may be through alternate mechanisms such as ER store-depletion or TCR signaling. Supporting this hypothesis, Jha et al. (2009) recently found Ca_V_1.4 to be localized to lipid rafts in the plasma membrane of murine T cells. Ca_V_1.4 was found to be associated with components of the T cell signaling complex. Given its location, Ca_V_1 channel activity could be regulated in T cells by downstream TCR signaling events.

Recent *in vivo* studies have directly addressed the controversy regarding the importance of voltage-dependent Ca^2+^ channels in T cell function. Mice with targeted deletions in the regulatory β subunits that mediate Ca_V_ channel assembly, plasma membrane targeting, and activation have been described (Badou et al., 2006; Buraei and Yang, 2010). The β3 and β4 family members are expressed in naïve CD4^+^ T cells and upregulated in activated T cells. Upon TCR cross-linking, CD4^+^ T cells from β3 or β4-deficient mice showed impaired Ca^2+^ influx, NFAT nuclear translocation, and cytokine secretion (Badou et al., 2006). Cav1.1 expression was found to be reduced in the β4-deficient T cells providing a possible role for Ca_V_1 in lymphocyte function (Badou et al., 2006). The same group later examined CD8^+^ T cell populations in a β3-deficient mouse (Jha et al., 2009). β3^−*/*−^ mice have reduced numbers of CD8^+^ T cells possibly due to increased spontaneous apoptosis induced by higher expression of Fas. Upon activation, these CD8^+^ T cells have decreased Ca^2+^ entry, proliferation, and NFAT nuclear translocation. β3 was found to associate with Ca_V_1.4 and several TCR signaling proteins suggesting its role in TCR gated Ca^2+^ signaling (Jha et al., 2009). Similarly, when the AHNAK1 protein, a large scaffold protein required for Ca_V_1.1 surface expression, was disrupted, T cells had reduced Ca^2+^ influx and NFAT activation that equated to poor effector function (Matza et al., 2008, 2009). Recently, Cabral et al. (2010) began to address differential Ca^2+^ signaling in T cell subsets. This study demonstrated that Ca_V_1.2 and Ca_V_1.3 channels were expressed in Th2 but not Th1 differentiated effector T cells. Knockdown of Ca_V_1.2 and/or Ca_V_1.3 expression in Th2 cells with antisense oligodeoxynucleotides resulted in reduced Ca^2+^ influx following TCR stimulation and impaired cytokine secretion (Cabral et al., 2010). In addition, Th2 cells with disrupted Ca_V_1 expression were impaired in their ability to induce asthma in an adoptive transfer model (Cabral et al., 2010). Further studies defining the Cav1 channel subtype or splice variant essential to various stages of development and activation of the T cell subsets will likely provide an explanation for differences in Ca^2+^ responses.

Omilusik et al. (2011) used a murine model deficient for Ca_V_1.4 (Mansergh et al., 2005), one of the pore-forming subunits of a Ca_V_ channel, to unequivocally establish a T cell-intrinsic role for Ca_V_1s in the activation, survival, and maintenance of naïve CD4^+^ and CD8^+^ T cells *in vivo*. Ca_V_1.4 was shown to be essential for TCR-induced regulation of cytosolic free Ca^2+^ and downstream TCR signaling, impacting activation of the Ras/ERK and NFAT pathways, IL-7 receptor expression and IL-7 responsiveness. The loss of Ca_V_1.4 and subsequently naïve peripheral T cells resulted in deficient immune responses when challenged with the model bacteria, *L. monocytogenes*. Instead of being activated by Ca^2+^ store release as in the case of ORAI1, it appears that Ca_V_1.4 may operate to create intracellular Ca^2+^ stores in the ER. Low-level TCR signaling through interactions with self-antigens (i.e., self-peptides/self-MHC molecules) may result in Ca_V_1.4-mediated Ca^2+^ influx from outside the cell, allowing the filling of intracellular stores and the initiation of a pro-survival program. This recent data supports the concept that in the absence of Ca_V_1.4, there is a reduction in the influx of extracellular Ca^2+^ coupled to self/MHC-TCR interaction, resulting in low cytoplasmic Ca^2+^ levels and depleted Ca^2+^ ER stores (Omilusik et al., 2011). Therefore, when Ca_V_1.4-deficient T cells are stimulated through the TCR, there is a defective Ca^2+^ release from the ER as a result of lower levels of stored Ca^2+^, decreased subsequent SOCE, and diminished inward Ca^2+^ flux through CRAC channels leading to weakened Ca^2+^-dependent signaling. Overall, the absence of tonic survival signals provided by Ca_V_1.4 results in failure of naïve T cells to thrive and perpetuates a state of immunological activation and exhaustion (Omilusik et al., 2011). Studies on other immune cells support this contention. For example, Ca_V_1.2 expressed in mast cells has been reported to protect against antigen-induced cell death by maintaining mitochondria integrity and inhibiting the mitochondrial cell death pathway (Suzuki et al., 2009). Using pharmacological agents and siRNA specific knockdown, Suzuki et al. (2009) demonstrated that Ca_V_1.2 channels protect mast cells from undergoing apoptosis following FcεRI activation as discerned by assessing mitochondrial membrane potential, cytochrome *c* release, and caspase-3/7 activation. Furthermore, though it remains unclear, it appears that Ca^2+^ influx through Ca_V_1.2 at the plasma membrane may be important for maintenance of the mitochondrial Ca^2+^ concentration, thereby providing the cell with pro-survival signals (Suzuki et al., 2009). In conclusion, it is of importance to note that knock-outs of the components of Ca_V_1 channels in T cells have, by large, more severe phenotypes than those of other categories of Ca^2+^ channels in T cells and, certainly, this argues strongly that Ca_V_1 channels play a significant role in regulating and orchestrating T cell biology.

It is interesting to consider and likely profoundly important for integrating the multiple functions of T cells with other homeostatic processes, that Ca_V_1 coexist in excitable and non-excitable cells with other Ca^2+^ channels and the interplay between the channels all likely contribute to the highly regulated Ca^2+^ signaling system. Ca_V_1 channels have been shown to interact with the ER/sarcoplasmic reticulum (SR) ryanodine receptors (RyRs) in excitable cells (Lanner et al., 2010). In skeletal muscle, Ca_V_1.1 channels are activated by membrane depolarization and through a physical interaction with RyR1 stimulate the release of Ca^2+^ from the SR. Similarly, in cardiac muscle, Ca_V_1.2 is triggered to mediate entry of extracellular Ca^2+^ which in turn activates RyR2 channels to release intracellular Ca^2+^ stores (Lanner et al., 2010). Both mechanisms have also been observed in neurons (Chavis et al., 1996; Mouton et al., 2001). Although T cells express RyRs (Hosoi et al., 2001) and these receptors have been shown to contribute to Ca^2+^ signaling following TCR activation (Hohenegger et al., 1999; Schwarzmann et al., 2002; Conrad et al., 2004), further studies are needed to demonstrate a Ca_V_1–RyR interaction.

An interplay between voltage-gated sodium channels (VGSC) and Ca_V_1 has also been suggested to shape the T cell Ca^2+^ signal. In excitable cells such as muscle and neurons, membrane depolarization by VGSC leads to an increase in cytosolic Ca^2+^ through the activation of Ca_V_ channels (Dravid et al., 2004; Fekete et al., 2009; Catterall, 2010). A recent study in T cells has determined an essential role for a VGSC in positive selection (Lo et al., 2012). Pharmacological inhibition and shRNA-mediated knockdown was used to demonstrate that the VGSC composed of a pore-forming SCN5a and a regulatory SCN5b subunit is necessary for Ca^2+^ influx during positive selection of CD4^+^ T cells. It is proposed that this SCN5a-SCN5b channel is expressed in double positive T cells in order to convert a weak positive selection signal into a sustained Ca^2+^ flux necessary for positive selection to take place. However, once in the periphery, T cells no longer express the channel to eliminate the possibility of autoimmunity (Lo et al., 2012). ORAI1 and STIM1 do not seem to contribute to thymic development of conventional TCRαβ T cells (Oh-Hora et al., 2013); therefore, it is an interesting idea that VGSC activation by kinases downstream of the TCR (Rook et al., 2012) can induce Ca^2+^ signaling by Ca_V_1 in developing T cells. Further studies are required to formally demonstrate a functional link between Ca_V_1 and VGSC channels in lymphocytes.

Recently, an interesting reciprocal relationship between Ca_V_1.2 and ORAI1 has been described (Park et al., 2010; Wang et al., 2010). After Ca^2+^ store depletion in the ER, STIM1 oligomers form at ER-plasma membrane junctions allowing the STIM1 CRAC-activating domain (CAD) to interact with the C-terminus of ORAI1 and Ca_V_1.2 channels. ORAI1 channels are activated by STIM1 and subsequently open causing sustained Ca^2+^ influx from the extracellular space. Conversely, STIM1 inhibits Ca^2+^ influx through Ca_V_1.2 and promotes its internalization, further shutting down the activity of the channel (Park et al., 2010; Wang et al., 2010). It is interesting to speculate that strong TCR signaling through engagement by a foreign peptide-MHC may trigger this activation of ORAI1 and inhibition of Ca_V_1 channels (Figure 1). However, low-level TCR signaling through interactions with self-antigens (i.e., self-peptides/self-MHC molecules) may not induce STIM1 to localize to the plasma membrane thereby activating Ca_V_ and co-ordinately inhibiting ORAI1. This results in Ca_V_1-mediated Ca^2+^ influx from outside the cell, filling of depleted intracellular stores, and induction of a signaling cascade to activate a pro-survival program within the naïve T cell. The activation and inhibition of Ca_V_1 channels through STIM1 or other TCR-mediated events is an intriguing concept and will likely be the focus of many new studies.

Although Ca_V_1 function is vital for T cell Ca^2+^ signaling, their specific functions have yet to be fully explored. Further work is required to clarify the role played by each Ca_V_1 channel family member as well as the other Ca^2+^ channels in shaping the Ca^2+^ signal. Altogether, these studies do provide a new framework for understanding the regulation of lymphocyte biology through the function of several Ca^2+^ channels, particularly the L-type Ca^2+^ channels, in the storage of intracellular Ca^2+^ and operative Ca^2+^ regulation during antigen receptor-mediated signal transduction.

Overall, the translational aspects of the current research in the field of Ca^2+^ channel biology have direct implications in designing new modalities for modifying T cell responses using drugs that are known to control Ca^2+^ channels activities, such as the plethora of drugs that already exist for modifying Ca_V_1 channels. Agents that target the Ca_V_1 splice variants expressed in lymphocytes and inhibit the activity of the channel may serve as more specific immunosuppressants than the current options. Relevant applications for these agents may include therapy for autoimmune diseases, reduction of transplant rejection risk, and treatment of other disorders requiring suppression or in the case of existing immunodeficiency, activation of the immune system.

## Conflict of Interest Statement

The authors declare that the research was conducted in the absence of any commercial or financial relationships that could be construed as a potential conflict of interest.
